# Supplementary material for: Levels of Circulating IgM and IgY Natural Antibodies in Broiler Chicks: Association with Genotype and Farming Systems
Source: Biology (Basel). 2023 Feb 14;12(2):304. doi: 10.3390/biology12020304 (PMC9952908; doi:10.3390/biology12020304)
Supplement: Supplementary file 1 [file biology-12-00304-s001.zip › Table S1.pdf]

Table S1. Chemical composition[1] of the free-range chickens' feed.

	Starter (1 – 17 days)		Grower (18 – 35 days)		Finisher (36 days - slaughter)	
**Metabolic Energy (kcal/kg)**	3029.82		3080.0		3080.0	
**Fibers (%)**	2.54		2.46		2.46	
**Calcium (%)**	1.00		0.90		0.90	
**Phosphorus (%)**	0.68		0.63		0.63	
**Sodium (%)**	0.18		0.17		0.17	
**Cloride (%)**	0.22		0.21		0.21	
**Potassium (%)**	0.91		0.84		0.84	
**Amino Acids**	**Total**	**Digest**	**Total**	**Digest**	**Total**	**Digest**
**Lysine (%)**	1.42	1.30	1.20	1.09	1.20	1.09
**Methionine (%)**	0.76	0.73	0.61	0.58	0.61	0.58
**Methionine + Cysteine (%)**	1.13	1.03	0.95	0.85	0.95	0.85
**Threonine (%)**	0.99	0.87	0.80	0.69	0.80	0.69
**Cystine (%)**	0.34		0.32		0.32	

[1]Chemical composition was noted according to feed formulation and NIR tests for verification
