# Supplementary material for: Levels of Circulating IgM and IgY Natural Antibodies in Broiler Chicks: Association with Genotype and Farming Systems
Source: Biology (Basel). 2023 Feb 14;12(2):304. doi: 10.3390/biology12020304 (PMC9952908; doi:10.3390/biology12020304)
Supplement: Supplementary file 1 [file biology-12-00304-s001.zip › Table S2.pdf]

Table S2. Chemical composition[1] of the conventional chickens' feed.

	Starter (0 – 10 days)		Grower (11 -24 days)		Finisher 1 (25 – 39 days)		Finisher 2 (40 days – slaughter)	
**Metabolic Energy (kcal/kg)**	3000		3100		3200		3200	
**Fibers (%)**	2.4		2.4		2.3		2.2	
**Calcium (%)**	0.96		0.87		0.78		0.75	
**Phosphorus (%)**	0.48		0.44		0.39		0.38	
**Sodium (%)**	0.16-0.23		0.16-0.23		0.16-0.20		0.16-0.20	
**Cloride (%)**	0.16-0.23		0.16-0.23		0.16-0.23		0.16-0.23	
**Potassium (%)**	0.40-1.00		-		-		-	
**Amino Acids**	**Total**	**Digest**	**Total**	**Digest**	**Total**	**Digest**	**Total**	**Digest**
**Lysine (%)**	1.44	1.28	1.29	1.15	1.15	1.02	1.08	0.96
**Methionine +Cysteine (%)**	1.08	0.95	0.99	0.87	0.90	0.80	0.85	0.75
**Methionine (%)**	0.56	0.51	0.51	0.47	0.47	0.43	0.44	0.40
**Threonine (%)**	0.97	0.86	0.88	0.77	0.78	0.68	0.73	0.64
**Valine (%)**	1.10	0.96	1.00	0.87	0.89	0.78	0.84	0.73
**Isoleukine (%)**	0.97	0.86	0.89	0.78	0.80	0.70	0.75	0.66
**Arginine (%)**	1.52	1.37	1.37	1.37	1.21	1.09	1.14	1.03
**Tryptophan (%)**	0.23	0.20	0.21	0.18	0.18	0.16	0.17	0.15
**Leucine (%)**	1.58	1.41	1.42	1.27	1.26	1.12	1.19	1.06

[1]Chemical composition was noted according to feed formulation and NIR tests for verification
