# Supplementary material for: Levels of Circulating IgM and IgY Natural Antibodies in Broiler Chicks: Association with Genotype and Farming Systems
Source: Biology (Basel). 2023 Feb 14;12(2):304. doi: 10.3390/biology12020304 (PMC9952908; doi:10.3390/biology12020304)
Supplement: Supplementary file 1 [file biology-12-00304-s001.zip › Table S3.pdf]

**Table S3**. Chemical analysis of the three different animal feeds.

| | Protein% | Fat% | Moisture% | Ash% |
|---|---|---|---|---|
| **Feed A** | 21,6 | 5,1 | 8,9 | 2,6 |
| **Feed B** | 21,5 | 5,2 | 9,2 | 2,2 |
| **Feed C** | 20,9 | 5,1 | 9,2 | 2,7 |

Feed A, Feed of the free range-chickens; Feed B, Feed of the free-range chickens dietary supplemented with a premix of oregano and oregano oil; Feed C, Feed of the conventional chickens.
