# Supplementary material for: Levels of Circulating IgM and IgY Natural Antibodies in Broiler Chicks: Association with Genotype and Farming Systems
Source: Biology (Basel). 2023 Feb 14;12(2):304. doi: 10.3390/biology12020304 (PMC9952908; doi:10.3390/biology12020304)
Supplement: Supplementary file 1 [file biology-12-00304-s001.zip › Table S4.pdf]

Date of placement	Date of slaughter	Production duration (d)	Genotype	Samples (Serum)	Slaughtered chickens (Industry)	Loses% (Mean)+2%	Total weight (Kg)	Mean weight (Kg)	Paste total	FCR	EPEF
19/10/2018	5/12/2018	47	ROSS 308 (C group)	15	44951	3.36	118958	2.646	215390	1.81	301
7/1/2019	21/2/2019			15	31172	4.5	68954	2.212	113360	1.64	286
25/3/2019	9/5/2019			15	29050	5.07	76111	2.62	137490	1.81	306
14/6/2019	31/7/2019			15	34690	2.83	91110	2.626	164570	1.81	301
27/8/2019	16/10/2019			15	13568	3.61	38531	2.84	68140	1.77	310
21/11/2019	11/1/2020			15	24852	2.54	76299	3.07	135390	1.77	331
18/2/2020	4/4/2020			15	29231	4.47	79051	2.704	136690	1.73	325
11/5/2020	25/6/2020			15	25030	3.01	63300	2.529	106850	1.69	323
3/8/2020	15/9/2020			15	14640	4.31	37243	2.544	67290	1.81	313
14/10/2020	30/11/2020			15	15376	2.75	42728	2.779	75780	1.77	324
26/1/2021	11/3/2021			15	20861	4.43	51529	2.47	88620	1.72	312
5/8/2021	18/09/21			15	21440	9.6	55311	2.58	93340	1.69	326
19/10/2021	29/11/21			15	12840	1.6	31680	2.467	56860	1.79	325
25/01/2022	10/3/2022			15	21900	5	50747	2.317	85310	1.68	300
28/9/2018	5/12/2018	67	SASSO (FR group)	15	6302	1.93	14830	2.353	35850	2.42	140
14/12/2018	21/2/2019			15	5920	3.27	13474	2.276	33830	2.51	127
8/3/2019	9/5/2019			15	5696	7.7	13860	2.433	33300	2.4	151
22/5/2019	31/7/2019			15	6216	4.78	14791	2.38	37670	2.55	127
9/8/2019	16/10/2019			15	5552	9.28	14642	2.637	35550	2.43	145
6/11/2019	12/1/2020			15	6960	5.23	17315	2.488	42040	2.43	145
28/1/2020	3/4/2020			15	6944	2.75	16661	2.399	39530	2.37	149
21/4/2020	25/6/2020			15	5726	4.85	14384	2.512	34060	2.37	155
8/7/2020	15/9/2020			15	5822	4.87	14038	2.411	34240	2.44	136
22/9/2020	30/11/2020			15	5968	2.48	15285	2.561	38500	2.52	144
1/1/2021	11/3/2021			15	6770	5.18	19330	2.855	47850	2.48	159
12/7/2021	20/9/2021			15	5760	5.88	13840	2.403	32360	2.34	138
29/9/2021	7/12/2021			15	5816	4.97	15255	2.623	37540	2.46	147
4/1/2022	10/3/2022			15	5760	1.45	13464	2.338	32640	2.42	146
14/12/2018	21/2/2019	67	SASSO (FRp group)	15	2825	7.68	7300	2.584	18650	2.55	135
8/3/2019	9/5/2019			15	2870	6.21	6910	2.408	16280	2.36	155
6/11/2019	10/1/2020			15	2944	3.79	7420	2.52	18670	2.52	148
28/1/2020	2/4/2020			15	2960	3.27	6900	2.331	16836	2.44	142
21/4/2020	24/6/2020			15	2912	4.84	7170	2.462	17280	2.41	152
8/7/2020	15/9/2020			15	2868	6.27	6920	2.413	17023	2.46	133
22/9/2020	30/11/2020			15	2960	3.27	7370	2.49	18572	2.52	139
1/1/2021	11/3/2021			15	2972	2.88	7770	2.614	19270	2.48	148
12/7/2021	18/9/2021			15	2768	9.54	6550	2.366	16720	2.55	123

FCR: Feed Conversion Ratio

EPEF: European Production Efficiency Factor $EPEF = \frac{BW\ (kg)\ x\ Liveability\ (\%)}{FCR\ x\ slaughter\ age\ (d)} \times 100$
