# Supplementary material for: Levels of Circulating IgM and IgY Natural Antibodies in Broiler Chicks: Association with Genotype and Farming Systems
Source: Biology (Basel). 2023 Feb 14;12(2):304. doi: 10.3390/biology12020304 (PMC9952908; doi:10.3390/biology12020304)
Supplement: Supplementary file 1 [file biology-12-00304-s001.zip › Table S5.pdf]

Regression type: Least squares

Dependent variable		IgM anti-ACT					
Analysis of Variance		SS	DF	MS	F (DFn, DFd)	P value	
Regression		6.488	12	0.5407	F (12, 28) = 2.497	P=0.0226	
Residual		6.062	28	0.2165			
Total		12.55	40				
Parameter estimates	Variable	Estimate	Standard error	95% CI (asymptotic)	|t|	P value	P value summary
β0	Intercept	-0.4245	3.747	-8.100 to 7.251	0.1133	0.9106	ns
β1	Season[3]	3.187	4.948	-6.948 to 13.32	0.6442	0.5247	ns
β2	Season[1]	-1.615	9.527	-21.13 to 17.90	0.1695	0.8666	ns
β3	Season[2]	5	5.393	-6.047 to 16.05	0.9271	0.3618	ns
β4	Breed[1]	-1.689	3.47	-8.798 to 5.420	0.4867	0.6303	ns
β5	EPEF	0.003844	0.01217	-0.02109 to 0.02878	0.3158	0.7545	ns
β6	Season[3] : Breed[1]	-1.876	2.732	-7.473 to 3.720	0.6867	0.4979	ns
β7	Season[1] : Breed[1]	0.2276	5.049	-10.11 to 10.57	0.04508	0.9644	ns
β8	Season[2] : Breed[1]	-2.483	3.013	-8.654 to 3.688	0.8242	0.4168	ns
β9	Season[3] : EPEF	-0.009867	0.0158	-0.04223 to 0.02249	0.6246	0.5373	ns
β10	Season[1] : EPEF	0.007686	0.03027	-0.05432 to 0.06970	0.2539	0.8014	ns
β11	Season[2] : EPEF	-0.0144	0.01719	-0.04960 to 0.02081	0.8377	0.4093	ns
β12	Breed[1] : EPEF	0.02179	0.01387	-0.006609 to 0.05020	1.572	0.1272	ns
Goodness of Fit							
Degrees of Freedom		28					
R squared		0.517					
Multicollinearity	Variable	VIF	R2 with other variables				
β0	Intercept						
β1	Season[3]	959.8	0.999				
β2	Season[1]	2147	0.9995				
β3	Season[2]	1081	0.9991				
β4	Breed[1]	512.9	0.9981				
β5	EPEF	189.4	0.9947				
β6	Season[3] : Breed[1]	222	0.9955				
β7	Season[1] : Breed[1]	425	0.9976				
β8	Season[2] : Breed[1]	243.4	0.9959				
β9	Season[3] : EPEF	482.6	0.9979				
β10	Season[1] : EPEF	1065	0.9991				
β11	Season[2] : EPEF	561.8	0.9982				
β12	Breed[1] : EPEF	167.4	0.994				
Normality of Residuals		Statistics	P value	Passed normality test (alpha=0.05)?	P value summary		
Anderson-Darling (A2*)		0.1826	0.9056	Yes	ns		
D'Agostino-Pearson omnibus (K2)		0.6835	0.7105	Yes	ns		
Shapiro-Wilk (W)		0.9903	0.9765	Yes	ns		
Kolmogorov-Smirnov (distance)		0.07685	>0.1000	Yes	ns		
Rows in table		45					
Rows skipped (missing data)		4					
Rows analyzed (# cases)		41					
Number of parameter estimates		13					
#cases/#parameters		3.2					

Dependent variable		IgM anti-DNA					
Analysis of Variance		SS	DF	MS	F (DFn, DFd)	P value	
Regression		5.582	12	0.4652	F (12, 28) = 2.388	P=0.0283	
Residual		5.454	28	0.1948			
Total		11.04	40				
Parameter estimates	Variable	Estimate	Standard error	95% CI (asymptotic)	|t|	P value	P value summary
β0	Intercept	2.308	3.554	-4.972 to 9.589	0.6495	0.5213	ns
β1	Season[3]	1.553	4.693	-8.060 to 11.17	0.3309	0.7432	ns
β2	Season[1]	-9.303	9.037	-27.81 to 9.207	1.03	0.312	ns
β3	Season[2]	0.2907	5.116	-10.19 to 10.77	0.05682	0.9551	ns
β4	Breed[1]	-2.775	3.292	-9.517 to 3.968	0.8429	0.4064	ns
β5	EPEF	-0.003962	0.01155	-0.02761 to 0.01969	0.3432	0.734	ns
β6	Season[3] : Breed[1]	-0.6387	2.591	-5.947 to 4.670	0.2465	0.8071	ns
β7	Season[1] : Breed[1]	4.712	4.789	-5.098 to 14.52	0.9839	0.3336	ns
β8	Season[2] : Breed[1]	0.2897	2.858	-5.564 to 6.143	0.1014	0.92	ns
β9	Season[3] : EPEF	-0.00507	0.01498	-0.03576 to 0.02562	0.3383	0.7376	ns
β10	Season[1] : EPEF	0.03234	0.02871	-0.02648 to 0.09115	1.126	0.2696	ns
β11	Season[2] : EPEF	0.0005138	0.0163	-0.03288 to 0.03390	0.03152	0.9751	ns
β12	Breed[1] : EPEF	0.01781	0.01315	-0.009134 to 0.04475	1.354	0.1866	ns
Goodness of Fit							
Degrees of Freedom		28					
R squared		0.5058					
Multicollinearity	Variable	VIF	R2 with other variables				
β0	Intercept						
β1	Season[3]	959.8	0.999				
β2	Season[1]	2147	0.9995				
β3	Season[2]	1081	0.9991				
β4	Breed[1]	512.9	0.9981				
β5	EPEF	189.4	0.9947				
β6	Season[3] : Breed[1]	222	0.9955				
β7	Season[1] : Breed[1]	425	0.9976				
β8	Season[2] : Breed[1]	243.4	0.9959				
β9	Season[3] : EPEF	482.6	0.9979				
β10	Season[1] : EPEF	1065	0.9991				
β11	Season[2] : EPEF	561.8	0.9982				
β12	Breed[1] : EPEF	167.4	0.994				
Normality of Residuals		Statistics	P value	Passed normality test (alpha=0.05)?	P value summary		
Anderson-Darling (A2*)		0.3451	0.4683	Yes	ns		
D'Agostino-Pearson omnibus (K2)		2.057	0.3575	Yes	ns		
Shapiro-Wilk (W)		0.9798	0.6673	Yes	ns		
Kolmogorov-Smirnov (distance)		0.09317	>0.1000	Yes	ns		
Rows in table		45					
Rows skipped (missing data)		4					
Rows analyzed (# cases)		41					
Number of parameter estimates		13					
#cases/#parameters		3.2					

Dependent variable		IgM anti-TNP					
Analysis of Variance		SS	DF	MS	F (DFn, DFd)	P value	
Regression		5.328	12	0.444	F (12, 28) = 2.145	P=0.0472	
Residual		5.796	28	0.207			
Total		11.12	40				
Parameter estimates	Variable	Estimate	Standard error	95% CI (asymptotic)	|t|	P value	P value summary
β0	Intercept	-0.3746	3.664	-7.880 to 7.131	0.1022	0.9193	ns
β1	Season[3]	3.008	4.838	-6.902 to 12.92	0.6218	0.5391	ns
β2	Season[1]	-12.93	9.316	-32.01 to 6.153	1.388	0.1761	ns
β3	Season[2]	4.6	5.273	-6.202 to 15.40	0.8723	0.3904	ns
β4	Breed[1]	0.07704	3.393	-6.874 to 7.028	0.0227	0.982	ns
β5	EPEF	0.004469	0.0119	-0.01991 to 0.02885	0.3755	0.7101	ns
β6	Season[3] : Breed[1]	-1.693	2.671	-7.165 to 3.779	0.6338	0.5314	ns
β7	Season[1] : Breed[1]	6.449	4.937	-3.664 to 16.56	1.306	0.2021	ns
β8	Season[2] : Breed[1]	-2.521	2.946	-8.555 to 3.514	0.8556	0.3995	ns
β9	Season[3] : EPEF	-0.009561	0.01545	-0.04120 to 0.02208	0.619	0.5409	ns
β10	Season[1] : EPEF	0.04355	0.0296	-0.01708 to 0.1042	1.471	0.1524	ns
β11	Season[2] : EPEF	-0.01435	0.0168	-0.04877 to 0.02007	0.8539	0.4004	ns
β12	Breed[1] : EPEF	0.009788	0.01356	-0.01798 to 0.03756	0.7219	0.4763	ns
Goodness of Fit							
Degrees of Freedom		28					
R squared		0.479					
Multicollinearity	Variable	VIF	R2 with other variables				
β0	Intercept						
β1	Season[3]	959.8	0.999				
β2	Season[1]	2147	0.9995				
β3	Season[2]	1081	0.9991				
β4	Breed[1]	512.9	0.9981				
β5	EPEF	189.4	0.9947				
β6	Season[3] : Breed[1]	222	0.9955				
β7	Season[1] : Breed[1]	425	0.9976				
β8	Season[2] : Breed[1]	243.4	0.9959				
β9	Season[3] : EPEF	482.6	0.9979				
β10	Season[1] : EPEF	1065	0.9991				
β11	Season[2] : EPEF	561.8	0.9982				
β12	Breed[1] : EPEF	167.4	0.994				
Normality of Residuals		Statistics	P value	Passed normality test (alpha=0.05)?	P value summary		
Anderson-Darling (A2*)		0.3827	0.3821	Yes	ns		
D'Agostino-Pearson omnibus (K2)		2.463	0.2919	Yes	ns		
Shapiro-Wilk (W)		0.9715	0.3841	Yes	ns		
Kolmogorov-Smirnov (distance)		0.102	>0.1000	Yes	ns		
Rows in table		45					
Rows skipped (missing data)		4					
Rows analyzed (# cases)		41					
Number of parameter estimates		13					
#cases/#parameters		3.2					

Dependent variable		IgM anti-LPS					
Analysis of Variance		SS	DF	MS	F (DFn, DFd)	P value	
Regression		9.907	12	0.8256	F (12, 28) = 3.661	P=0.0023	
Residual		6.313	28	0.2255			
Total		16.22	40				
Parameter estimates	Variable	Estimate	Standard error	95% CI (asymptotic)	|t|	P value	P value summary
β0	Intercept	-8.582	3.824	-16.41 to -0.7490	2.244	0.0329	*
β1	Season[3]	7.325	5.049	-3.018 to 17.67	1.451	0.158	ns
β2	Season[1]	-12.5	9.723	-32.41 to 7.418	1.285	0.2092	ns
β3	Season[2]	12.89	5.504	1.618 to 24.17	2.342	0.0265	*
β4	Breed[1]	6.403	3.542	-0.8518 to 13.66	1.808	0.0814	ns
β5	EPEF	0.0309	0.01242	0.005458 to 0.05635	2.488	0.0191	*
β6	Season[3] : Breed[1]	-3.694	2.788	-9.406 to 2.017	1.325	0.1959	ns
β7	Season[1] : Breed[1]	5.972	5.153	-4.583 to 16.53	1.159	0.2562	ns
β8	Season[2] : Breed[1]	-7.093	3.074	-13.39 to -0.7949	2.307	0.0287	*
β9	Season[3] : EPEF	-0.02488	0.01612	-0.05790 to 0.008146	1.543	0.134	ns
β10	Season[1] : EPEF	0.0396	0.03089	-0.02368 to 0.1029	1.282	0.2104	ns
β11	Season[2] : EPEF	-0.04185	0.01754	-0.07778 to -0.005928	2.386	0.024	*
β12	Breed[1] : EPEF	-0.003691	0.01415	-0.03268 to 0.02529	0.2609	0.7961	ns
Goodness of Fit							
Degrees of Freedom		28					
R squared		0.6108					
Multicollinearity	Variable	VIF	R2 with other variables				
β0	Intercept						
β1	Season[3]	959.8	0.999				
β2	Season[1]	2147	0.9995				
β3	Season[2]	1081	0.9991				
β4	Breed[1]	512.9	0.9981				
β5	EPEF	189.4	0.9947				
β6	Season[3] : Breed[1]	222	0.9955				
β7	Season[1] : Breed[1]	425	0.9976				
β8	Season[2] : Breed[1]	243.4	0.9959				
β9	Season[3] : EPEF	482.6	0.9979				
β10	Season[1] : EPEF	1065	0.9991				
β11	Season[2] : EPEF	561.8	0.9982				
β12	Breed[1] : EPEF	167.4	0.994				
Normality of Residuals		Statistics	P value	Passed normality test (alpha=0.05)?	P value summary		
Anderson-Darling (A2*)		0.3109	0.5391	Yes	ns		
D'Agostino-Pearson omnibus (K2)		0.1712	0.918	Yes	ns		
Shapiro-Wilk (W)		0.9806	0.6998	Yes	ns		
Kolmogorov-Smirnov (distance)		0.09922	>0.1000	Yes	ns		
Rows in table		45					
Rows skipped (missing data)		4					
Rows analyzed (# cases)		41					
Number of parameter estimates		13					
#cases/#parameters		3.2					

Dependent variable		IgY anti-ACT					
Analysis of Variance		SS	DF	MS	F (DFn, DFd)	P value	
Regression		3.017	12	0.2514	F (12, 28) = 1.022	P=0.4560	
Residual		6.885	28	0.2459			
Total		9.901	40				
Parameter estimates	Variable	Estimate	Standard error	95% CI (asymptotic)	|t|	P value	P value summary
β0	Intercept	-8.226	3.993	-16.41 to -0.04598	2.06	0.0488	*
β1	Season[3]	7.338	5.273	-3.463 to 18.14	1.392	0.175	ns
β2	Season[1]	19.4	10.15	-1.400 to 40.19	1.911	0.0664	ns
β3	Season[2]	7.176	5.747	-4.597 to 18.95	1.249	0.2222	ns
β4	Breed[1]	6.225	3.698	-1.350 to 13.80	1.683	0.1034	ns
β5	EPEF	0.03152	0.01297	0.004952 to 0.05809	2.43	0.0218	*
β6	Season[3] : Breed[1]	-4.076	2.912	-10.04 to 1.888	1.4	0.1725	ns
β7	Season[1] : Breed[1]	-10.64	5.381	-21.66 to 0.3865	1.977	0.058	ns
β8	Season[2] : Breed[1]	-3.76	3.211	-10.34 to 2.817	1.171	0.2515	ns
β9	Season[3] : EPEF	-0.02506	0.01683	-0.05955 to 0.009422	1.489	0.1477	ns
β10	Season[1] : EPEF	-0.06192	0.03226	-0.1280 to 0.004164	1.919	0.0652	ns
β11	Season[2] : EPEF	-0.0237	0.01831	-0.06121 to 0.01382	1.294	0.2062	ns
β12	Breed[1] : EPEF	-0.00643	0.01478	-0.03670 to 0.02384	0.4352	0.6668	ns
Goodness of Fit							
Degrees of Freedom		28					
R squared		0.3047					
Multicollinearity	Variable	VIF	R2 with other variables				
β0	Intercept						
β1	Season[3]	959.8	0.999				
β2	Season[1]	2147	0.9995				
β3	Season[2]	1081	0.9991				
β4	Breed[1]	512.9	0.9981				
β5	EPEF	189.4	0.9947				
β6	Season[3] : Breed[1]	222	0.9955				
β7	Season[1] : Breed[1]	425	0.9976				
β8	Season[2] : Breed[1]	243.4	0.9959				
β9	Season[3] : EPEF	482.6	0.9979				
β10	Season[1] : EPEF	1065	0.9991				
β11	Season[2] : EPEF	561.8	0.9982				
β12	Breed[1] : EPEF	167.4	0.994				
Normality of Residuals		Statistics	P value	Passed normality test (alpha=0.05)?	P value summary		
Anderson-Darling (A2*)		0.2809	0.6237	Yes	ns		
D'Agostino-Pearson omnibus (K2)		1.365	0.5052	Yes	ns		
Shapiro-Wilk (W)		0.9782	0.6077	Yes	ns		
Kolmogorov-Smirnov (distance)		0.1012	>0.1000	Yes	ns		
Rows in table		45					
Rows skipped (missing data)		4					
Rows analyzed (# cases)		41					
Number of parameter estimates		13					
#cases/#parameters		3.2					

Dependent variable		IgY anti-DNA					
Analysis of Variance		SS	DF	MS	F (DFn, DFd)	P value	
Regression		6.225	12	0.5188	F (12, 28) = 6.785	P<0.0001	
Residual		2.141	28	0.07646			
Total		8.366	40				
Parameter estimates	Variable	Estimate	Standard error	95% CI (asymptotic)	|t|	P value	P value summary
β0	Intercept	-3.239	2.227	-7.800 to 1.323	1.454	0.157	ns
β1	Season[3]	3.487	2.94	-2.536 to 9.511	1.186	0.2456	ns
β2	Season[1]	-4.158	5.662	-15.76 to 7.439	0.7345	0.4688	ns
β3	Season[2]	0.6086	3.205	-5.957 to 7.174	0.1899	0.8508	ns
β4	Breed[1]	2.661	2.062	-1.564 to 6.885	1.29	0.2075	ns
β5	EPEF	0.01534	0.007234	0.0005192 to 0.03015	2.12	0.043	*
β6	Season[3] : Breed[1]	-1.638	1.624	-4.964 to 1.688	1.009	0.3217	ns
β7	Season[1] : Breed[1]	2.121	3.001	-4.026 to 8.267	0.7068	0.4856	ns
β8	Season[2] : Breed[1]	0.01642	1.79	-3.651 to 3.684	0.009173	0.9927	ns
β9	Season[3] : EPEF	-0.01226	0.009388	-0.03149 to 0.006974	1.306	0.2023	ns
β10	Season[1] : EPEF	0.01486	0.01799	-0.02199 to 0.05171	0.8262	0.4157	ns
β11	Season[2] : EPEF	-0.001589	0.01021	-0.02251 to 0.01933	0.1556	0.8775	ns
β12	Breed[1] : EPEF	-0.005908	0.00824	-0.02279 to 0.01097	0.717	0.4793	ns
Goodness of Fit							
Degrees of Freedom		28					
R squared		0.7441					
Multicollinearity	Variable	VIF	R2 with other variables				
β0	Intercept						
β1	Season[3]	959.8	0.999				
β2	Season[1]	2147	0.9995				
β3	Season[2]	1081	0.9991				
β4	Breed[1]	512.9	0.9981				
β5	EPEF	189.4	0.9947				
β6	Season[3] : Breed[1]	222	0.9955				
β7	Season[1] : Breed[1]	425	0.9976				
β8	Season[2] : Breed[1]	243.4	0.9959				
β9	Season[3] : EPEF	482.6	0.9979				
β10	Season[1] : EPEF	1065	0.9991				
β11	Season[2] : EPEF	561.8	0.9982				
β12	Breed[1] : EPEF	167.4	0.994				
Normality of Residuals		Statistics	P value	Passed normality test (alpha=0.05)?	P value summary		
Anderson-Darling (A2*)		0.2867	0.6052	Yes	ns		
D'Agostino-Pearson omnibus (K2)		1.349	0.5095	Yes	ns		
Shapiro-Wilk (W)		0.9695	0.3329	Yes	ns		
Kolmogorov-Smirnov (distance)		0.07374	>0.1000	Yes	ns		
Rows in table		45					
Rows skipped (missing data)		4					
Rows analyzed (# cases)		41					
Number of parameter estimates		13					
#cases/#parameters		3.2					

Dependent variable		IgY anti-TNP					
Analysis of Variance		SS	DF	MS	F (DFn, DFd)	P value	
Regression		1.731	12	0.1442	F (12, 28) = 0.9247	P=0.5366	
Residual		4.367	28	0.156			
Total		6.098	40				
Parameter estimates	Variable	Estimate	Standard error	95% CI (asymptotic)	|t|	P value	P value summary
β0	Intercept	-6.671	3.18	-13.19 to -0.1561	2.097	0.0451	*
β1	Season[3]	5.549	4.2	-3.054 to 14.15	1.321	0.1971	ns
β2	Season[1]	-2.483	8.087	-19.05 to 14.08	0.307	0.7611	ns
β3	Season[2]	1.786	4.578	-7.591 to 11.16	0.3902	0.6993	ns
β4	Breed[1]	6.553	2.946	0.5194 to 12.59	2.225	0.0343	*
β5	EPEF	0.02457	0.01033	0.003406 to 0.04573	2.378	0.0245	*
β6	Season[3] : Breed[1]	-3.032	2.319	-7.782 to 1.718	1.308	0.2017	ns
β7	Season[1] : Breed[1]	0.9565	4.286	-7.822 to 9.735	0.2232	0.825	ns
β8	Season[2] : Breed[1]	-0.9407	2.557	-6.179 to 4.297	0.3679	0.7157	ns
β9	Season[3] : EPEF	-0.01791	0.01341	-0.04538 to 0.009555	1.336	0.1924	ns
β10	Season[1] : EPEF	0.008944	0.02569	-0.04369 to 0.06158	0.3481	0.7304	ns
β11	Season[2] : EPEF	-0.005752	0.01459	-0.03563 to 0.02413	0.3943	0.6963	ns
β12	Breed[1] : EPEF	-0.01659	0.01177	-0.04070 to 0.007517	1.41	0.1696	ns
Goodness of Fit							
Degrees of Freedom		28					
R squared		0.2838					
Multicollinearity	Variable	VIF	R2 with other variables				
β0	Intercept						
β1	Season[3]	959.8	0.999				
β2	Season[1]	2147	0.9995				
β3	Season[2]	1081	0.9991				
β4	Breed[1]	512.9	0.9981				
β5	EPEF	189.4	0.9947				
β6	Season[3] : Breed[1]	222	0.9955				
β7	Season[1] : Breed[1]	425	0.9976				
β8	Season[2] : Breed[1]	243.4	0.9959				
β9	Season[3] : EPEF	482.6	0.9979				
β10	Season[1] : EPEF	1065	0.9991				
β11	Season[2] : EPEF	561.8	0.9982				
β12	Breed[1] : EPEF	167.4	0.994				
Normality of Residuals		Statistics	P value	Passed normality test (alpha=0.05)?	P value summary		
Anderson-Darling (A2*)		0.6256	0.0963	Yes	ns		
D'Agostino-Pearson omnibus (K2)		12.72	0.0017	No	**		
Shapiro-Wilk (W)		0.9301	0.0145	No	*		
Kolmogorov-Smirnov (distance)		0.1024	>0.1000	Yes	ns		
Rows in table		45					
Rows skipped (missing data)		4					
Rows analyzed (# cases)		41					
Number of parameter estimates		13					
#cases/#parameters		3.2					

Dependent variable		IgY anti-LPS					
Analysis of Variance		SS	DF	MS	F (DFn, DFd)	P value	
Regression		4.614	12	0.3845	F (12, 28) = 1.980	P=0.0669	
Residual		5.438	28	0.1942			
Total		10.05	40				
Parameter estimates	Variable	Estimate	Standard error	95% CI (asymptotic)	|t|	P value	P value summary
β0	Intercept	-7.999	3.549	-15.27 to -0.7291	2.254	0.0322	*
β1	Season[3]	15.98	4.686	6.380 to 25.58	3.41	0.002	**
β2	Season[1]	1.724	9.023	-16.76 to 20.21	0.1911	0.8498	ns
β3	Season[2]	13.32	5.108	2.852 to 23.78	2.607	0.0145	*
β4	Breed[1]	3.707	3.287	-3.025 to 10.44	1.128	0.2689	ns
β5	EPEF	0.02944	0.01153	0.005830 to 0.05306	2.554	0.0164	*
β6	Season[3] : Breed[1]	-8.571	2.588	-13.87 to -3.271	3.312	0.0026	**
β7	Season[1] : Breed[1]	-1.594	4.782	-11.39 to 8.202	0.3333	0.7414	ns
β8	Season[2] : Breed[1]	-7.356	2.853	-13.20 to -1.511	2.578	0.0155	*
β9	Season[3] : EPEF	-0.05113	0.01496	-0.08178 to -0.02048	3.417	0.002	**
β10	Season[1] : EPEF	-0.005254	0.02867	-0.06398 to 0.05347	0.1833	0.8559	ns
β11	Season[2] : EPEF	-0.04322	0.01628	-0.07656 to -0.009876	2.655	0.0129	*
β12	Breed[1] : EPEF	0.01054	0.01313	-0.01636 to 0.03744	0.8028	0.4289	ns
Goodness of Fit							
Degrees of Freedom		28					
R squared		0.459					
Multicollinearity	Variable	VIF	R2 with other variables				
β0	Intercept						
β1	Season[3]	959.8	0.999				
β2	Season[1]	2147	0.9995				
β3	Season[2]	1081	0.9991				
β4	Breed[1]	512.9	0.9981				
β5	EPEF	189.4	0.9947				
β6	Season[3] : Breed[1]	222	0.9955				
β7	Season[1] : Breed[1]	425	0.9976				
β8	Season[2] : Breed[1]	243.4	0.9959				
β9	Season[3] : EPEF	482.6	0.9979				
β10	Season[1] : EPEF	1065	0.9991				
β11	Season[2] : EPEF	561.8	0.9982				
β12	Breed[1] : EPEF	167.4	0.994				
Normality of Residuals		Statistics	P value	Passed normality test (alpha=0.05)?	P value summary		
Anderson-Darling (A2*)		0.472	0.232	Yes	ns		
D'Agostino-Pearson omnibus (K2)		5.817	0.0546	Yes	ns		
Shapiro-Wilk (W)		0.9615	0.1781	Yes	ns		
Kolmogorov-Smirnov (distance)		0.1138	>0.1000	Yes	ns		
Rows in table		45					
Rows skipped (missing data)		4					
Rows analyzed (# cases)		41					
Number of parameter estimates		13					
#cases/#parameters		3.2					

EPEF: European Production Efficiency Factor
Season[1] : Spring
Season[2] : Summer
Season[3] : Automn
Breed[1] : Slow growth
