# Supplementary material for: Levels of Circulating IgM and IgY Natural Antibodies in Broiler Chicks: Association with Genotype and Farming Systems
Source: Biology (Basel). 2023 Feb 14;12(2):304. doi: 10.3390/biology12020304 (PMC9952908; doi:10.3390/biology12020304)
Supplement: Supplementary file 1 [file biology-12-00304-s001.zip › Table S6.pdf]

MV: Multiple linear regression		IgM anti-ACT						
Dependent variable	**EPEF**							
Regression type	Least squares							
Analysis of Variance		SS	DF	MS	F (DFn, DFd)	P value		
Regression		272185	12	22682	F (12, 28) = 136.9	P<0.0001		
Residual		4640	28	165.7				
Total		276826	40					
Parameter estimates	Variable	Estimate	Standard error	95% CI (asymptotic)	|t|	P value	P value summary	
β0	Intercept	301.9	13.36	274.6 to 329.3	22.61	<0.0001	****	
β1	IgM ACT	7.008	15.46	-24.67 to 38.68	0.4532	0.6539	ns	
β2	Season[3]	15.05	13	-11.58 to 41.68	1.158	0.2568	ns	
β3	Season[1]	15.16	30.8	-47.94 to 78.25	0.492	0.6265	ns	
β4	Season[2]	12.28	19.97	-28.63 to 53.19	0.6149	0.5436	ns	
β5	Breed[1]	-184.6	15.15	-215.6 to -153.6	12.18	<0.0001	****	
β6	IgM ACT : Season[3]	-9.313	11.81	-33.50 to 14.87	0.7888	0.4369	ns	
β7	IgM ACT : Season[1]	-8.011	18.72	-46.35 to 30.33	0.428	0.6719	ns	
β8	IgM ACT : Season[2]	-5.784	17.17	-40.95 to 29.38	0.3369	0.7387	ns	
β9	IgM ACT : Breed[1]	9.091	12.97	-17.48 to 35.66	0.7009	0.4892	ns	
β10	Season[3] : Breed[1]	-1.035	14.05	-29.81 to 27.74	0.0737	0.9418	ns	
β11	Season[1] : Breed[1]	4.84	18.67	-33.41 to 43.09	0.2592	0.7974	ns	
β12	Season[2] : Breed[1]	-12.04	19.29	-51.56 to 27.48	0.6242	0.5375	ns	
Goodness of Fit								
Degrees of Freedom		28						
R squared		0.9832						
Multicollinearity	Variable	VIF	R2 with other variables					
β0	Intercept							
β1	IgM ACT	18.11	0.9448					
β2	Season[3]	8.654	0.8844					
β3	Season[1]	29.33	0.9659					
β4	Season[2]	19.37	0.9484					
β5	Breed[1]	12.77	0.9217					
β6	IgM ACT : Season[3]	14.9	0.9329					
β7	IgM ACT : Season[1]	26.26	0.9619					
β8	IgM ACT : Season[2]	45.12	0.9778					
β9	IgM ACT : Breed[1]	31.12	0.9679					
β10	Season[3] : Breed[1]	7.667	0.8696					
β11	Season[1] : Breed[1]	7.596	0.8683					
β12	Season[2] : Breed[1]	13.04	0.9233					
Normality of Residuals		Statistics	P value	Passed normality test (alpha=0.05)?	P value summary			
Anderson-Darling (A2*)		0.1794	0.9111	Yes	ns			
D'Agostino-Pearson omnibus (K2)		0.3179	0.853	Yes	ns			
Shapiro-Wilk (W)		0.9829	0.7847	Yes	ns			
Kolmogorov-Smirnov (distance)		0.05839	>0.1000	Yes	ns			
Rows in table		45						
Rows skipped (missing data)		4						
Rows analyzed (# cases)		41						
Number of parameter estimates		13						
#cases/#parameters		3.2						

MV: Multiple linear regression		IgM anti-DNA						
Dependent variable	**EPEF**							
Regression type	Least squares							
Analysis of Variance		SS	DF	MS	F (DFn, DFd)	P value		
Regression		271872	12	22656	F (12, 28) = 128.1	P<0.0001		
Residual		4953	28	176.9				
Total		276826	40					
Parameter estimates	Variable	Estimate	Standard error	95% CI (asymptotic)	|t|	P value	P value summary	
β0	Intercept	314.9	23.03	267.7 to 362.0	13.67	<0.0001	****	
β1	IgM ACT	-6.97	20.21	-48.36 to 34.42	0.3449	0.7327	ns	
β2	Season[3]	6.046	18.27	-31.39 to 43.48	0.3309	0.7432	ns	
β3	Season[1]	0.1721	37.28	-76.20 to 76.55	0.004615	0.9964	ns	
β4	Season[2]	8.325	22.54	-37.86 to 54.51	0.3693	0.7147	ns	
β5	Breed[1]	-181.3	21.06	-224.4 to -138.1	8.607	<0.0001	****	
β6	IgM ACT : Season[3]	1.165	14.42	-28.38 to 30.71	0.08079	0.9362	ns	
β7	IgM ACT : Season[1]	7.212	18.9	-31.51 to 45.93	0.3816	0.7057	ns	
β8	IgM ACT : Season[2]	2.051	15.1	-28.87 to 32.97	0.1358	0.8929	ns	
β9	IgM ACT : Breed[1]	12.49	17.24	-22.82 to 47.81	0.7247	0.4746	ns	
β10	Season[3] : Breed[1]	-9.921	13.68	-37.94 to 18.10	0.7254	0.4743	ns	
β11	Season[1] : Breed[1]	-7.894	21	-50.90 to 35.12	0.3759	0.7098	ns	
β12	Season[2] : Breed[1]	-20.05	15.91	-52.65 to 12.54	1.26	0.218	ns	
Goodness of Fit								
Degrees of Freedom		28						
R squared		0.9821						
Multicollinearity	Variable	VIF	R2 with other variables					
β0	Intercept							
β1	IgM ACT	25.47	0.9607					
β2	Season[3]	16.02	0.9376					
β3	Season[1]	40.25	0.9752					
β4	Season[2]	23.12	0.9568					
β5	Breed[1]	23.11	0.9567					
β6	IgM ACT : Season[3]	25.6	0.9609					
β7	IgM ACT : Season[1]	37.94	0.9736					
β8	IgM ACT : Season[2]	39.87	0.9749					
β9	IgM ACT : Breed[1]	59.47	0.9832					
β10	Season[3] : Breed[1]	6.808	0.8531					
β11	Season[1] : Breed[1]	8.996	0.8888					
β12	Season[2] : Breed[1]	8.308	0.8796					
Normality of Residuals		Statistics	P value	Passed normality test (alpha=0.05)?	P value summary			
Anderson-Darling (A2*)		0.2749	0.6436	Yes	ns			
D'Agostino-Pearson omnibus (K2)		0.3159	0.8539	Yes	ns			
Shapiro-Wilk (W)		0.9843	0.834	Yes	ns			
Kolmogorov-Smirnov (distance)		0.08846	>0.1000	Yes	ns			
Rows in table		45						
Rows skipped (missing data)		4						
Rows analyzed (# cases)		41						
Number of parameter estimates		13						
#cases/#parameters		3.2						

MV: Multiple linear regression		IgM anti-TNP						
Dependent variable	**EPEF**							
Regression type	Least squares							
Analysis of Variance		SS	DF	MS	F (DFn, DFd)	P value		
Regression		271962	12	22663	F (12, 28) = 130.5	P<0.0001		
Residual		4864	28	173.7				
Total		276826	40					
Parameter estimates	Variable	Estimate	Standard error	95% CI (asymptotic)	|t|	P value	P value summary	
β0	Intercept	296.7	18.11	259.6 to 333.8	16.38	<0.0001	****	
β1	IgM ACT	10.58	16.89	-24.03 to 45.18	0.6261	0.5363	ns	
β2	Season[3]	16.89	16.24	-16.38 to 50.15	1.04	0.3073	ns	
β3	Season[1]	-12.44	31.31	-76.57 to 51.69	0.3973	0.6941	ns	
β4	Season[2]	21.98	18	-14.89 to 58.85	1.221	0.2321	ns	
β5	Breed[1]	-173.7	19.32	-213.3 to -134.2	8.993	<0.0001	****	
β6	IgM ACT : Season[3]	-9.193	13.18	-36.19 to 17.81	0.6975	0.4913	ns	
β7	IgM ACT : Season[1]	6.361	18.65	-31.83 to 44.55	0.3411	0.7355	ns	
β8	IgM ACT : Season[2]	-13.22	14.53	-42.98 to 16.54	0.9097	0.3707	ns	
β9	IgM ACT : Breed[1]	0.394	14.78	-29.88 to 30.67	0.02666	0.9789	ns	
β10	Season[3] : Breed[1]	-1.821	14.68	-31.90 to 28.26	0.124	0.9022	ns	
β11	Season[1] : Breed[1]	6.694	19.6	-33.45 to 46.84	0.3416	0.7352	ns	
β12	Season[2] : Breed[1]	-3.241	15.4	-34.79 to 28.31	0.2104	0.8349	ns	
Goodness of Fit								
Degrees of Freedom		28						
R squared		0.9824						
Multicollinearity	Variable	VIF	R2 with other variables					
β0	Intercept							
β1	IgM ACT	18.28	0.9453					
β2	Season[3]	12.89	0.9224					
β3	Season[1]	28.9	0.9654					
β4	Season[2]	15.01	0.9334					
β5	Breed[1]	19.81	0.9495					
β6	IgM ACT : Season[3]	21.98	0.9545					
β7	IgM ACT : Season[1]	36.6	0.9727					
β8	IgM ACT : Season[2]	26.27	0.9619					
β9	IgM ACT : Breed[1]	42.51	0.9765					
β10	Season[3] : Breed[1]	7.991	0.8749					
β11	Season[1] : Breed[1]	7.981	0.8747					
β12	Season[2] : Breed[1]	7.927	0.8739					
Normality of Residuals		Statistics	P value	Passed normality test (alpha=0.05)?	P value summary			
Anderson-Darling (A2*)		0.1798	0.9106	Yes	ns			
D'Agostino-Pearson omnibus (K2)		0.05989	0.9705	Yes	ns			
Shapiro-Wilk (W)		0.9901	0.9739	Yes	ns			
Kolmogorov-Smirnov (distance)		0.07053	>0.1000	Yes	ns			
Rows in table		45						
Rows skipped (missing data)		4						
Rows analyzed (# cases)		41						
Number of parameter estimates		13						
#cases/#parameters		3.2						

MV: Multiple linear regression		IgM anti-LPS						
Dependent variable	**EPEF**							
Regression type	Least squares							
Analysis of Variance		SS	DF	MS	F (DFn, DFd)	P value		
Regression		273261	12	22772	F (12, 28) = 178.9	P<0.0001		
Residual		3565	28	127.3				
Total		276826	40					
Parameter estimates	Variable	Estimate	Standard error	95% CI (asymptotic)	|t|	P value	P value summary	
β0	Intercept	280.7	10.54	259.1 to 302.3	26.64	<0.0001	****	
β1	IgM ACT	29.07	9.745	9.103 to 49.03	2.982	0.0059	**	
β2	Season[3]	22.99	10.57	1.339 to 44.64	2.175	0.0382	*	
β3	Season[1]	12.02	16.08	-20.91 to 44.95	0.7475	0.461	ns	
β4	Season[2]	33.3	12.69	7.302 to 59.31	2.624	0.0139	*	
β5	Breed[1]	-157.9	12.99	-184.6 to -131.3	12.15	<0.0001	****	
β6	IgM ACT : Season[3]	-11.46	8.747	-29.38 to 6.455	1.31	0.2007	ns	
β7	IgM ACT : Season[1]	-9.503	12.06	-34.21 to 15.20	0.7879	0.4374	ns	
β8	IgM ACT : Season[2]	-27.03	11.37	-50.31 to -3.754	2.379	0.0244	*	
β9	IgM ACT : Breed[1]	-17.7	9.643	-37.46 to 2.051	1.836	0.077	ns	
β10	Season[3] : Breed[1]	-4.581	13.37	-31.96 to 22.80	0.3427	0.7344	ns	
β11	Season[1] : Breed[1]	12.11	12.89	-14.30 to 38.51	0.9394	0.3555	ns	
β12	Season[2] : Breed[1]	6.779	13.11	-20.08 to 33.64	0.5169	0.6093	ns	
Goodness of Fit								
Degrees of Freedom		28						
R squared		0.9871						
Multicollinearity	Variable	VIF	R2 with other variables					
β0	Intercept							
β1	IgM ACT	12.1	0.9174					
β2	Season[3]	7.448	0.8657					
β3	Season[1]	10.4	0.9038					
β4	Season[2]	10.19	0.9018					
β5	Breed[1]	12.23	0.9182					
β6	IgM ACT : Season[3]	13.83	0.9277					
β7	IgM ACT : Season[1]	10.49	0.9046					
β8	IgM ACT : Season[2]	17.25	0.942					
β9	IgM ACT : Breed[1]	23.82	0.958					
β10	Season[3] : Breed[1]	9.038	0.8894					
β11	Season[1] : Breed[1]	4.711	0.7877					
β12	Season[2] : Breed[1]	7.84	0.8725					
Normality of Residuals		Statistics	P value	Passed normality test (alpha=0.05)?	P value summary			
Anderson-Darling (A2*)		0.3269	0.5096	Yes	ns			
D'Agostino-Pearson omnibus (K2)		3.213	0.2006	Yes	ns			
Shapiro-Wilk (W)		0.9786	0.621	Yes	ns			
Kolmogorov-Smirnov (distance)		0.08465	>0.1000	Yes	ns			
Rows in table		45						
Rows skipped (missing data)		4						
Rows analyzed (# cases)		41						
Number of parameter estimates		13						
#cases/#parameters		3.2						

MV: Multiple linear regression		IgY anti-ACT						
Dependent variable	**EPEF**							
Regression type	Least squares							
Analysis of Variance		SS	DF	MS	F (DFn, DFd)	P value		
Regression		272859	12	22738	F (12, 28) = 160.5	P<0.0001		
Residual		3967	28	141.7				
Total		276826	40					
Parameter estimates	Variable	Estimate	Standard error	95% CI (asymptotic)	|t|	P value	P value summary	
β0	Intercept	269	14.51	239.3 to 298.7	18.53	<0.0001	****	
β1	IgY ACT	26.21	9.069	7.633 to 44.79	2.89	0.0074	**	
β2	Season[3]	35.2	17.05	0.2767 to 70.12	2.065	0.0483	*	
β3	Season[1]	47.84	20.31	6.235 to 89.45	2.355	0.0257	*	
β4	Season[2]	33.66	17.02	-1.211 to 68.53	1.977	0.0579	ns	
β5	Breed[1]	-151.6	14.86	-182.0 to -121.2	10.2	<0.0001	****	
β6	IgY ACT : Season[3]	-16.78	11.69	-40.72 to 7.152	1.436	0.162	ns	
β7	IgY ACT : Season[1]	-27.05	11.48	-50.56 to -3.545	2.357	0.0256	*	
β8	IgY ACT : Season[2]	-16.99	10.29	-38.06 to 4.081	1.652	0.1098	ns	
β9	IgY ACT : Breed[1]	-10.52	8.666	-28.27 to 7.234	1.214	0.235	ns	
β10	Season[3] : Breed[1]	-10.21	10.74	-32.22 to 11.79	0.9507	0.3499	ns	
β11	Season[1] : Breed[1]	-1.615	12.98	-28.20 to 24.97	0.1245	0.9018	ns	
β12	Season[2] : Breed[1]	-11.25	10.55	-32.87 to 10.36	1.067	0.2953	ns	
Goodness of Fit								
Degrees of Freedom		28						
R squared		0.9857						
Multicollinearity	Variable	VIF	R2 with other variables					
β0	Intercept							
β1	IgY ACT	5.747	0.826					
β2	Season[3]	17.41	0.9426					
β3	Season[1]	14.91	0.933					
β4	Season[2]	16.46	0.9392					
β5	Breed[1]	14.37	0.9304					
β6	IgY ACT : Season[3]	13.88	0.928					
β7	IgY ACT : Season[1]	10.79	0.9073					
β8	IgY ACT : Season[2]	16.16	0.9381					
β9	IgY ACT : Breed[1]	13.5	0.9259					
β10	Season[3] : Breed[1]	5.244	0.8093					
β11	Season[1] : Breed[1]	4.292	0.767					
β12	Season[2] : Breed[1]	4.561	0.7807					
Normality of Residuals		Statistics	P value	Passed normality test (alpha=0.05)?	P value summary			
Anderson-Darling (A2*)		0.2539	0.7153	Yes	ns			
D'Agostino-Pearson omnibus (K2)		0.5159	0.7726	Yes	ns			
Shapiro-Wilk (W)		0.9864	0.8971	Yes	ns			
Kolmogorov-Smirnov (distance)		0.07695	>0.1000	Yes	ns			
Rows in table		45						
Rows skipped (missing data)		4						
Rows analyzed (# cases)		41						
Number of parameter estimates		13						
#cases/#parameters		3.2						

MV: Multiple linear regression		IgY anti-DNA						
Dependent variable	**EPEF**							
Regression type	Least squares							
Analysis of Variance		SS	DF	MS	F (DFn, DFd)	P value		
Regression		272777	12	22731	F (12, 28) = 157.2	P<0.0001		
Residual		4049	28	144.6				
Total		276826	40					
Parameter estimates	Variable	Estimate	Standard error	95% CI (asymptotic)	|t|	P value	P value summary	
β0	Intercept	247.4	26.26	193.7 to 301.2	9.423	<0.0001	****	
β1	IgY ACT	40.58	17.35	5.049 to 76.12	2.339	0.0267	*	
β2	Season[3]	50.83	27.52	-5.543 to 107.2	1.847	0.0753	ns	
β3	Season[1]	-25.57	57.04	-142.4 to 91.27	0.4483	0.6574	ns	
β4	Season[2]	32.04	29.91	-29.23 to 93.31	1.071	0.2933	ns	
β5	Breed[1]	-118.7	24.5	-168.9 to -68.54	4.846	<0.0001	****	
β6	IgY ACT : Season[3]	-26.87	19.9	-67.63 to 13.90	1.35	0.1878	ns	
β7	IgY ACT : Season[1]	3.348	28.06	-54.13 to 60.82	0.1193	0.9059	ns	
β8	IgY ACT : Season[2]	-19.39	18.37	-57.01 to 18.24	1.056	0.3002	ns	
β9	IgY ACT : Breed[1]	-23.26	16.48	-57.02 to 10.50	1.411	0.1691	ns	
β10	Season[3] : Breed[1]	-30.21	14.62	-60.16 to -0.2612	2.066	0.0482	*	
β11	Season[1] : Breed[1]	25.23	32.08	-40.49 to 90.95	0.7864	0.4382	ns	
β12	Season[2] : Breed[1]	-20.71	15.13	-51.69 to 10.27	1.369	0.1818	ns	
Goodness of Fit								
Degrees of Freedom		28						
R squared		0.9854						
Multicollinearity	Variable	VIF	R2 with other variables					
β0	Intercept							
β1	IgY ACT	17.41	0.9426					
β2	Season[3]	44.46	0.9775					
β3	Season[1]	115.2	0.9913					
β4	Season[2]	49.81	0.9799					
β5	Breed[1]	38.28	0.9739					
β6	IgY ACT : Season[3]	25.05	0.9601					
β7	IgY ACT : Season[1]	63.76	0.9843					
β8	IgY ACT : Season[2]	37.86	0.9736					
β9	IgY ACT : Breed[1]	19.23	0.948					
β10	Season[3] : Breed[1]	9.52	0.895					
β11	Season[1] : Breed[1]	25.7	0.9611					
β12	Season[2] : Breed[1]	9.185	0.8911					
Normality of Residuals		Statistics	P value	Passed normality test (alpha=0.05)?	P value summary			
Anderson-Darling (A2*)		0.6051	0.1084	Yes	ns			
D'Agostino-Pearson omnibus (K2)		5.422	0.0665	Yes	ns			
Shapiro-Wilk (W)		0.9572	0.1252	Yes	ns			
Kolmogorov-Smirnov (distance)		0.1093	>0.1000	Yes	ns			
Rows in table		45						
Rows skipped (missing data)		4						
Rows analyzed (# cases)		41						
Number of parameter estimates		13						
#cases/#parameters		3.2						

MV: Multiple linear regression		IgY anti-TNP						
Dependent variable	**EPEF**							
Regression type	Least squares							
Analysis of Variance		SS	DF	MS	F (DFn, DFd)	P value		
Regression		273149	12	22762	F (12, 28) = 173.3	P<0.0001		
Residual		3677	28	131.3				
Total		276826	40					
Parameter estimates	Variable	Estimate	Standard error	95% CI (asymptotic)	|t|	P value	P value summary	
β0	Intercept	275.4	11.39	252.1 to 298.8	24.17	<0.0001	****	
β1	IgY ACT	36.23	11.22	13.25 to 59.21	3.23	0.0032	**	
β2	Season[3]	33.38	13.65	5.423 to 61.34	2.446	0.021	*	
β3	Season[1]	13.37	23.09	-33.92 to 60.66	0.5792	0.5671	ns	
β4	Season[2]	12.75	16.67	-21.40 to 46.90	0.7647	0.4509	ns	
β5	Breed[1]	-142.8	11.89	-167.2 to -118.5	12.01	<0.0001	****	
β6	IgY ACT : Season[3]	-29.89	11.94	-54.35 to -5.434	2.503	0.0184	*	
β7	IgY ACT : Season[1]	-17.43	16.55	-51.34 to 16.48	1.053	0.3015	ns	
β8	IgY ACT : Season[2]	-10.16	14.56	-39.99 to 19.68	0.6973	0.4914	ns	
β9	IgY ACT : Breed[1]	-27.09	10.51	-48.62 to -5.554	2.577	0.0155	*	
β10	Season[3] : Breed[1]	-4.265	10.02	-24.78 to 16.25	0.4259	0.6735	ns	
β11	Season[1] : Breed[1]	10.58	14.33	-18.78 to 39.94	0.7381	0.4666	ns	
β12	Season[2] : Breed[1]	-6.589	10.21	-27.49 to 14.32	0.6457	0.5238	ns	
Goodness of Fit								
Degrees of Freedom		28						
R squared		0.9867						
Multicollinearity	Variable	VIF	R2 with other variables					
β0	Intercept							
β1	IgY ACT	5.844	0.8289					
β2	Season[3]	12.04	0.9169					
β3	Season[1]	20.79	0.9519					
β4	Season[2]	17.04	0.9413					
β5	Breed[1]	9.93	0.8993					
β6	IgY ACT : Season[3]	10.99	0.909					
β7	IgY ACT : Season[1]	14.28	0.93					
β8	IgY ACT : Season[2]	15.56	0.9357					
β9	IgY ACT : Breed[1]	10.52	0.905					
β10	Season[3] : Breed[1]	4.919	0.7967					
β11	Season[1] : Breed[1]	5.648	0.823					
β12	Season[2] : Breed[1]	4.604	0.7828					
Normality of Residuals		Statistics	P value	Passed normality test (alpha=0.05)?	P value summary			
Anderson-Darling (A2*)		0.2221	0.8174	Yes	ns			
D'Agostino-Pearson omnibus (K2)		0.8392	0.6573	Yes	ns			
Shapiro-Wilk (W)		0.9822	0.7575	Yes	ns			
Kolmogorov-Smirnov (distance)		0.07269	>0.1000	Yes	ns			
Rows in table		45						
Rows skipped (missing data)		4						
Rows analyzed (# cases)		41						
Number of parameter estimates		13						
#cases/#parameters		3.2						

MV: Multiple linear regression		IgY anti-LPS						
Dependent variable	**EPEF**							
Regression type	Least squares							
Analysis of Variance		SS	DF	MS	F (DFn, DFd)	P value		
Regression		273145	12	22762	F (12, 28) = 173.1	P<0.0001		
Residual		3681	28	131.5				
Total		276826	40					
Parameter estimates	Variable	Estimate	Standard error	95% CI (asymptotic)	|t|	P value	P value summary	
β0	Intercept	285.6	11.12	262.8 to 308.4	25.67	<0.0001	****	
β1	IgY ACT	20.66	9.094	2.028 to 39.29	2.271	0.031	*	
β2	Season[3]	41.87	15.21	10.71 to 73.03	2.752	0.0103	*	
β3	Season[1]	10.91	17.76	-25.47 to 47.29	0.6144	0.5439	ns	
β4	Season[2]	36.16	16.75	1.849 to 70.47	2.159	0.0396	*	
β5	Breed[1]	-159.3	13.45	-186.9 to -131.8	11.84	<0.0001	****	
β6	IgY ACT : Season[3]	-31.49	11.31	-54.66 to -8.322	2.784	0.0095	**	
β7	IgY ACT : Season[1]	-6.666	10.86	-28.90 to 15.57	0.6141	0.5441	ns	
β8	IgY ACT : Season[2]	-26.86	14.91	-57.41 to 3.688	1.801	0.0825	ns	
β9	IgY ACT : Breed[1]	-9.372	10.02	-29.89 to 11.15	0.9355	0.3575	ns	
β10	Season[3] : Breed[1]	4.091	10.86	-18.15 to 26.33	0.3768	0.7092	ns	
β11	Season[1] : Breed[1]	7.333	12.85	-18.99 to 33.66	0.5706	0.5729	ns	
β12	Season[2] : Breed[1]	-5.677	10.81	-27.82 to 16.47	0.5251	0.6037	ns	
Goodness of Fit								
Degrees of Freedom		28						
R squared		0.9867						
Multicollinearity	Variable	VIF	R2 with other variables					
β0	Intercept							
β1	IgY ACT	6.324	0.8419					
β2	Season[3]	14.94	0.9331					
β3	Season[1]	12.29	0.9186					
β4	Season[2]	17.18	0.9418					
β5	Breed[1]	12.69	0.9212					
β6	IgY ACT : Season[3]	18.2	0.9451					
β7	IgY ACT : Season[1]	7.408	0.865					
β8	IgY ACT : Season[2]	18.83	0.9469					
β9	IgY ACT : Breed[1]	18.33	0.9454					
β10	Season[3] : Breed[1]	5.775	0.8268					
β11	Season[1] : Breed[1]	4.536	0.7795					
β12	Season[2] : Breed[1]	5.161	0.8062					
Normality of Residuals		Statistics	P value	Passed normality test (alpha=0.05)?	P value summary			
Anderson-Darling (A2*)		0.4572	0.2523	Yes	ns			
D'Agostino-Pearson omnibus (K2)		3.444	0.1787	Yes	ns			
Shapiro-Wilk (W)		0.9642	0.2206	Yes	ns			
Kolmogorov-Smirnov (distance)		0.09771	>0.1000	Yes	ns			
Rows in table		45						
Rows skipped (missing data)		4						
Rows analyzed (# cases)		41						
Number of parameter estimates		13						
#cases/#parameters		3.2						

EPEF: European Production Efficiency Factor
Season[1] : Spring
Season[2] : Summer
Season[3] : Automn
Breed[1] : Slow growth
