# Supplementary material for: Levels of Circulating IgM and IgY Natural Antibodies in Broiler Chicks: Association with Genotype and Farming Systems
Source: Biology (Basel). 2023 Feb 14;12(2):304. doi: 10.3390/biology12020304 (PMC9952908; doi:10.3390/biology12020304)
Supplement: Supplementary file 1 [file biology-12-00304-s001.zip › Table S7.pdf]

Principal Component Analysis	IgM C (fast growth broilers)					IgM FR (slow growth broilers)				
PC summary	PC1	PC2	PC3	PC4	PC5	PC1	PC2	PC3	PC4	PC5
Eigenvalue	2.912	1.104	0.6566	0.2007	0.1262	2.797	1.048	0.8895	0.2015	0.06437
Proportion of variance	58.25%	22.08%	13.13%	4.01%	2.52%	55.93%	20.96%	17.79%	4.03%	1.29%
Cumulative proportion of variance	58.25%	80.33%	93.46%	97.48%	100.00%	55.93%	76.89%	94.68%	98.71%	100.00%
Component selection	Selected	Selected				Selected	Selected			
Data summary										
Total number of variables	5					5				
Total number of components	5					5				
Component selection method	PCs with eigenvalues greater than 1.0 (“Kaiser rule”)					PCs with eigenvalues greater than 1.0 (“Kaiser rule”)				
Number of selected components	2					2				
Rows in table	14					14				
Rows skipped (missing data)	0					0				
Rows analyzed (# cases)	14					14				

Principal Component Analysis	IgY C (fast growth broilers)					IgY FR (slow growth broilers)				
PC summary	PC1	PC2	PC3	PC4	PC5	PC1	PC2	PC3	PC4	PC5
Eigenvalue	2.066	1.47	0.9446	0.3092	0.2096	2.009	1.397	0.7935	0.6426	0.1578
Proportion of variance	41.32%	29.41%	18.89%	6.18%	4.19%	40.19%	27.93%	15.87%	12.85%	3.16%
Cumulative proportion of variance	41.32%	70.73%	89.63%	95.81%	100.00%	40.19%	68.12%	83.99%	96.84%	100.00%
Component selection	Selected	Selected				Selected	Selected			
Data summary										
Total number of variables	5					5				
Total number of components	5					5				
Component selection method	PCs with eigenvalues greater than 1.0 (“Kaiser rule”)					PCs with eigenvalues greater than 1.0 (“Kaiser rule”)				
Number of selected components	2					2				
Rows in table	14					14				
Rows skipped (missing data)	0					0				
Rows analyzed (# cases)	14					14				
